# Pretreatment with a long-acting GnRH agonist for frozen-thawed embryo transfer cycles: how to improve live birth?

**DOI:** 10.1186/s13048-023-01277-0

**Published:** 2023-09-25

**Authors:** Bin Xu, Zhaojuan Hou, Nenghui Liu, Jing Zhao, Yanping Li

**Affiliations:** 1 Reproductive Medicine Center, Xiangya Hospital, Central South University, 87 Xiangya Road, Changsha City, Hunan Province People’s Republic of China 410008; 2 Clinical Research Center For Woman’s Reproductive Health in Hunan Province, Changsha City, China

**Keywords:** GnRH agonist, Follicular phase, Frozen-thawed embryo transfer, Hormone replacement treatment, Assisted reproductive technique

## Abstract

**Background:**

Whether pretreatment with gonadotropin-releasing hormone agonist (GnRHa) can improve the pregnancy outcomes in frozen-thawed embryo transfer (FET) cycles is controversial. The inconsistencies in the results of different studies would be related to the characteristics of the included patients and the protocol of GnRHa use. In this study, we investigated the efficacy of pretreatment with a long-acting GnRH agonist in the early follicular phase of FET cycles and determined which population was suitable for the protocol.

**Results:**

We retrospectively included 630 and 1141 patients in the GnRHa FET and hormone replacement treatment (HRT) FET without GnRHa groups respectively, between October 2017 and March 2019 at a university-affiliated in vitro fertilization center. On the second or third day of menstruation, 3.75 mg of leuprorelin was administered. After 14 days, HRT was initiated for endometrial preparation. No significant differences were observed between the two groups in terms of patient characteristics. However, the GnRHa FET group showed a higher percentage of endometrium with a triple line pattern (94.8% vs 89.6%, p < 0.001) on the day of progesterone administration, with increased implantation (35.6% vs 29.8%, *p* = 0.005), clinical pregnancy (49.8% vs 43.3%, *p* = 0.008), and live birth rate (39.4% vs 33.7%, *p* = 0.016), than the HRT FET cycles with similar endometrial thickness, ectopic pregnancy and early miscarriage rates. Binary logistic regression analysis showed the GnRHa FET group to be associated with an increased chance of clinical pregnancy (*P*=0.028, odds ratio [OR] 1.32, 95% confidence interval [CI] 1.03–1.70) and live birth (*P*=0.013, odds ratio [OR] 1.34, 95% confidence interval [CI] 1.06–1.70) compared to the HRT FET without GnRHa group. After subgroup analysis, we found that the GnRHa FET group showed a significantly higher live birth rate in the subgroups of age < 40 years, primary infertility, with polycystic ovary syndrome (PCOS), and irregular menstruation.

**Conclusions:**

Pretreatment with a long-acting GnRHa during the early follicular phase improved the live birth rate in FET cycles. Age < 40 years, primary infertility, PCOS, and irregular menstruation are effective indications for endometrial preparation with GnRHa pretreatment in FET cycles. However, further randomized controlled trials are required to verify these results.

**Supplementary Information:**

The online version contains supplementary material available at 10.1186/s13048-023-01277-0.

## Background

Frozen-thawed embryo transfer (FET) allows the generation of embryos by in vitro fertilization (IVF) or intracytoplasmic sperm injection (ICSI), which can then be frozen for transfer after several months or years [1]. FET has been found to increase the cumulative pregnancy rate after one cycle of ovarian stimulation and oocyte retrieval [2]. FET also reduces the risk of ovarian hyperstimulation syndrome. Embryos from freeze-all strategy cycles, such as mild controlled ovarian stimulation cycle, progestin-primed ovarian stimulation cycle, and cancelled fresh cycles resulting from other conditions, including those with extremely high levels of serum progesterone, can be transferred during FET cycles. In recent years, the rates of FET cycles in Europe increased from 28% in 2010 to 32.3% in 2011 [3]. However, there is little consensus regarding the most efficient method for endometrial preparation in FET cycles. Nature cycles are mainly used in ovulatory women, whereas hormone replacement therapy (HRT) cycles have been utilized in anovulatory patients or in time-controlled situations. In HRT FET, estrogen and progesterone are administered in a sequential regimen, and it aims to suppress the development of the dominant follicle and mimic the hormone exposure of the endometrium. Initially, estrogen was administered for more than 12 days to induce the endometrial proliferation. Progesterone was then administered to initiate secretory changes associated with the endometrium reaching its optimal thickness, as observed by ultrasound. Synchronously developed embryos were thawed and transferred [1].

Since 1989, to mimic the down-regulation procedure of fresh cycles, a gonadotropin-releasing hormone agonist (GnRHa) has been applied and started from the mid-luteal phase to downregulate pituitary GnRH receptors and prevent follicular growth. For the subsequent menstrual cycle, estrogen and progesterone are administered sequentially, and this cycle is denoted as the HRT cycle. In the HRT cycle without GnRHa, the development of dominant follicles are also suppressed, providing a more economical, convenient, and comfortable procedure for patients undergoing IVF, in addition to being less time consuming. Therefore, an HRT cycle of FET without GnRHa has become a common method for endometrial preparation in anovulatory patients. El-Toukhy et al. reported that HRT FET with daily short-acting GnRHa starting in the mid-luteal phase of the menstrual cycle achieved significantly higher clinical pregnancy and live birth rates than those without GnRHa suppression [4]. However, other studies [5–12] found different results with similar clinical pregnancy rates. A Cochrane review [2] reported that HRT alone was associated with a clinical pregnancy rate similar to that of HRT with GnRHa suppression. Recently, a randomized control trial (RCT) [13] reported that pretreatment with long-acting GnRHa after 5–7 days of oral dydrogesterone in patients with polycystic ovary syndrome (PCOS) did not improve the clinical pregnancy rate in the HRT FET cycle.

Obviously, whether GnRHa pretreatment can improve the pregnancy outcomes in FET cycles is controversial. GnRHa was administered during the mid-luteal phase as a pretreatment in most of the above studies. We propose that the inconsistency of results in different studies is related to the characteristics of the included patients and the protocol of GnRHa administration. Therefore, we investigated the efficacy of pretreatment with a long-acting GnRHa in the early follicular phase of frozen-thawed embryo transfer cycles and determined which population was applicable to the protocol.

## Results

### Patient characteristics

A total of 630 and 1141 patients were included in the GnRHa FET group and HRT FET without GnRHa groups, respectively. There was no difference between the two groups in terms of age, menstrual cycle (regular menstruation:22–34 days), duration of infertility, type of infertility, percentage of PCOS (diagnosis according to Rotterdam Criteria), diminished ovarian reserves (anti-Müllerian hormone < 1.2 ng/ml), endometriosis, scarred uterus (uterus after surgery such as cesarean section or intramural myoma), uterine malformation, or the grade and number of transferred embryos (Table 1).

**Table 1:** Characteristics of patients

Characteristics	GnRHa FET (*n* = 630)	HRT without GnRHa (*n* = 1141)	*P*
Age	32.1 ± 5.2	32.4 ± 5.3	0.306
Duration of infertility	4.8 ± 3.6	5.1 ± 3.9	0.127
Type of infertility			0.108
Primary	310/630 (49.2)	516/1141 (45.2)	
Secondary	320/630 (50.8)	516/1141 (54.8)	
Number of pregnancies	1.2 ± 1.4	1.2 ± 1.5	0.245
PCOS(%)	111 (17.6)	238 (20.9)	0.101
DOR(%)	86 (13.7)	183 (16.0)	0.180
Scarred uterus(%)	102 (16.2)	158 (13.8)	0.182
Endometriosis(%)	23 (3.7)	36 (3.2)	0.578
Number of transferred embryos	1.7 ± 0.4	1.7 ± 0.4	0.890
Grade of transferred embryos^a^			0.472
Cleavage I	160 (22.7)	277 (23.9)	
Cleavage II	331 (46.9)	548 (47.4)	
Blastocyst	215 (30.5)	332 (28.7)	

*GnRHa* Gonadotropin-releasing hormone agonist, *HRT* Hormone replacement treatment, *PCOS* Polycystic ovary syndrome, *DOR* Decreased ovarian reserve

^a^If transferred embryo including at least one grade I cleavage embryo, one or two grade II cleavage embryo, and one or two blastocysts were classified as Cleavage I, Cleavage II, and blastocyst respectively

### Outcomes

The GnRHa FET group produced a higher percentage of endometrium with a triple line pattern (94.8% vs 89.6%, *p* < 0.001) on the day of progesterone administration, as well as an increased implantation (35.6% vs 29.8%, *p* = 0.005), biochemical pregnancy (60.6% vs 54.3%, *p* = 0.009), clinical pregnancy (49.8% vs 43.3%, *p* = 0.008), and live birth rates (39.4% vs 33.7%, *p* = 0.016) compared to the HRT FET group. However, the endometrial thickness, ectopic pregnancy rate, and early miscarriage rate were similar Table 2.

**Table 2:** Outcome of FET with or without GnRHa

Outcome	GnRHa FET (*n* = 630)	HRT without GnRHa (*n* = 1141)	*P*
Endometrial thickness	9.4 ± 1.5	9.3 ± 1.5	0.215
Endometrial pattern			0.000
Triple line	597 (94.8)	1022 (89.6)	
No triple line	33 (5.2)	119 (10.4)	
Biochemical pregnancy (%)	382 (60.6)	619 (54.3)	0.009
Implantation rate (%)	390/1093 (35.6)	591/1983 (29.8)	0.005
Clinical pregnancy (%)	314 (49.8)	494 (43.3)	0.008
Ectopic pregnancy (%)	8 (1.3)	17 (1.5)^a^	0.707
Early miscarriage (%)	37/630 (5.9)	65/1141 (5.7)	0.879
Live birth (%)	248 (39.4)	384(33.7)	0.016

*FET* Frozen-thawed embryo transfer, *GnRHa* Gonadotropin-releasing hormone agonist, *HRT* Hormone replacement treatment

^a^Included two heterotopic pregnancies

Binary logistic regression analysis was performed to evaluate the effect of each variable on clinical pregnancy (Table 3). Age, PCOS, number of transferred embryos, grade of transferred embryos, endometrial thickness and GnRHa use, rather than different forms of estrogen use were the independent factors affecting clinical pregnancy and live birth. The GnRHa FET group was associated with an increased chance of clinical pregnancy (*P* = 0.028, odds ratio [OR] 1.32, 95% confidence interval [CI] 1.03–1.7) and live birth rates (*P* = 0.013, odds ratio [OR] 1.34, 95% confidence interval [CI] 1.06–1.7) compared to HRT FET without GnRHa group. The Hosmer–Lemeshow test showed that the final models of clinical pregnancy (*p* = 0.222) and live birth (*p* = 0.343) fit well.

**Table 3:** Multivariable logistic regression analysis on patient’s variable effect on clinical pregnancy and live birth

Covariate	Clinical pregnancy^a^		Live birth^b^	
	OR (95% CI)	*P*	OR (95% CI)	*P*
Age	0.92(0.90–0.94)	0.000	0.91(0.89–0.93)	0.000
PCOS				
YES	Reference		1.00	
NO	0.69(0.53–0.89)	0.005	0.75(0.58–0.97)	0.032
Endometriosis				
YES	1.00		1.00	
NO	1.83(1.01–3.33)	0.045	1.53(0.81–2.89)	0.181
Number of transferred embryos	2.09(1.62–2.70)	0.000	1.97(1.50–2.57)	0.000
Grade of transferred embryos		0.000		0.000
Cleavage	0.50(0.39–0.64)		0.53(0.41–0.68)	
Blastocyst	1.00		1.00	
Endometrial thickness	1.09(1.02–1.16)	0.011	1.07(1.00–1.15)	0.04
Endometrial pattern				
Triple line	1.38 (0.95–2.00)	0.082	1.29(0.87–1.9)	0.194
No triple line	1.00		1.00	
Form of estrogen administration		0.570		0.548
Without estrogen	1.00		1.00	
Estradiol gel	0.67 (0.32–1.38)	0.67	0.73 (0.35–1.52)	0.408
Estradiol valerate	0.66 (0.33–1.35)	0.261	0.75 (0.36–1.54)	0.44
Femoston	0.74 (0.35–1.56)	0.439	0.88 (0.41–1.87)	0.746
Protocol				
With GnRha	1.32(1.03–1.70)	0.028	1.34(1.06–1.70)	0.013
Without GnRha	1.00		1.00	

*OR* Odds ratio, *CI* Confidence interval, *PCOS* Polycystic ovary syndrome, *DOR* Decreased ovarian reserve, *GnRHa* Gonadotropin-releasing hormone agonist, *HRT* Hormone replacement treatment

^a^Hosmer–Lemeshow test, *p* = 0.222

^b^Hosmer–Lemeshow test, *p* = 0.343

Whether GnRHa pretreatment improves pregnancy outcomes in different patient populations remains unknown. Patients were divided into two subgroups according to age, type of infertility, PCOS and menstrual cycle. Subgroups analysis were performed between age < 40 years with ≥ 40 years, primary with secondary infertility, PCOS with no PCOS, and regular with irregular menstruation respectively. The results are summarized in Table 4. The percentage of tri-line endometrium on the day of progesterone administration of the GnRHa FET group was significantly higher than that in the HRT FET group in all subgroups, except for the subgroup of patients with regular menstruation and age ≥40 years. In the subgroup of age < 40 years rather than ≥ 40 years, the GnRHa FET group produced higher implantation (38.1% vs 32.3%, *p* = 0.002), biochemical pregnancy (63.7% vs 58.6%, *p* = 0.019), clinical pregnancy (53.4% vs 46.6%, *p* = 0.01), and live birth rates (42.4% vs 37%, *p* = 0.036) than the HRT FET group. In patients with primary infertility, rather than secondary infertility, the implantation, clinical pregnancy and live birth rates were higher in the GnRHa FET group than the HRT FET group (42.0% vs. 33.3%, *p* < 0.001; 58.1% vs. 48.6%, *p* = 0.009; and 47.7% vs. 38.2%, *p* = 0.007, respectively).

**Table 4:** Subgroup analysis of FET Outcomes with or without GnRHa

	Age (years)						Type of infertility					
	< 40			≥ 40			Primary			Secondary		
	GnRHa (*N* = 556)	Without GnRHa (*N* = 999)	*p*	GnRHa (*N* = 74)	Without GnRHa (*N* = 142)	*p*	GnRHa (N = 310)	Without GnRHa (*N* = 516)	*p*	GnRHa (*N* = 320)	Without GnRHa (*N* = 625)	*p*
Age (years)	30.8 ± 4.1	31.0 ± 4.0	0.426	41.5 ± 1.5	41.9 ± 1.6	0.14	30.2 ± 4.4	30.5 ± 4.4	0.318	33.8 ± 5.3	33.8 ± 5.4	0.932
Number of transferred embryos	1.7 ± 0.4	1.7 ± 0.4	0.881	1.6 ± 0.4	1.6 ± 0.4	0.426	1.7 ± 0.4	1.7 ± 0.4	0.856	1.7 ± 0.4	1.7 ± 0.4	0.856
Grade of transferred embryos^a^(%)			0.352			0.836			0.286			0.958
Cleavage I	116/556 (20.9)	239/999 (23.9)		22 (29.7)	37 (26.1)		63/310 (20.3)	125/516 (24.2)		75/320 (23.4)	151/625 (24.2)	
Cleavage II	252/556 (45.3)	445/999 (44.5)		47 (63.5)	94 (66.2)		144/310 (46.5)	242/516 (46.9)		155/320 (48.4)	297/625 (47.5)	
Blastocyst	188/556 (33.8)	315/999 (31.5)		5 (6.8)	11 (7.7)		103/310 (33.2)	149/516 (28.9)		90/320 (28.1)	177/625 (28.3)	
Endometrial thickness (mm)	9.4 ± 1.5	9.3 ± 1.5	0.193	9.0 ± 1.4	9.0 ± 1.3	0.935	9.5 ± 1.5	9.4 ± 1.4	0.498	9.2 ± 1.5	9.1 ± 1.5	0.400
Triple- line Endometrium (%)	531 (95.5)	893 (89.4)	0.000	66 (89.2)	129 (90.8)	0.697	296/310 (95.5)	472/516 (91.5)	0.029	301/320 (94.1)	550/625 (88.0)	0.003
Biochemical pregnancy rate (%)	354/556 (63.7)	575/999 (58.6)	0.019	28 (37.8)	44 (31)	0.311	210/310 (67.7)	315/516 (61.0)	0.053	172/320 (53.8)	304/625 (48.6)	0.137
Implantation rate (%)	371/972 (38.1)	564/1743 (32.3)	0.002	19/121 (15.7)	27/240 (11.2)	0.231	230/548 (42.0)	305/915 (33.3)	0.000	160/545 (29.4)	286/1068 (26.8)	0.273
Clinical pregnancy rate (%)	297 (53.4)	466 (46.6)	0.01	17 (23)	28 (19.7)	0.576	180/310 (58.1)	251/516 (48.6)	0.009	134/320 (41.9)	243/625 (38.9)	0.374
Ectopic pregnancy (%)	8 (1.4)	16 (1.6)	0.803	0	1 (0.7)	1.000	5/310 (1.6)	6/516 (1.2)	0.585	3/320 (0.9)	11/625 (1.8)	0.322
Early miscarriage (%)	32 (5.8)	53 (5.3)	0.708	5 (6.8)	12 (8.5)	0.661	17/310(5.5)	34/516 (6.6)	0.523	20 (6.3)	31 (5.0)	0.406
Live birth (%)	236 (42.4)	370(37)	0.036	12 (16.2)	14 (9.9)	0.173	148(47.7)	197(38.2)	0.007	100(31.3)	187(29.9)	0.674
	PCOS						Menstruation^b^					
	Yes			No			Regular			Irregular		
	GnRHa (*N* = 111)	Without GnRHa (*N* = 238)	*p*	GnRHa (*N* = 519)	Without GnRHa (*N* = 903)	*p*	GnRHa (*N* = 397)	Without GnRHa (*N* = 638)	*p*	GnRHa (*N* = 207)	Without GnRHa (*N* = 453)	*p*
Age(years)	29.8 ± 3.9	29.7 ± 4.0	0.801	32.5 ± 5.3	33 ± 5.3	0.098	33.0 ± 5.3	33.4 ± 5.5	0.213	30.1 ± 4.3	30.7 ± 4.3	0.156
Number of transferred embryos	1.7 ± 0.4	1.7 ± 0.4	0.574	1.7 ± 0.4	1.7 ± 0.4	0.725	1.7 ± 0.4	1.7 ± 0.4	0.480	1.7 ± 0.4	1.7 ± 0.4	0.584
Grade of transferred embryos^a^			0.613			0.543			0.294			0.943
Cleavage I	21 (18.9)	51 (21.4)		117 (22.5)	225 (24.9)		92 (23.2)	170 (26.6)		43 (20.8)	93 (20.5)	
Cleavage II	40/ (36.0)	93 (39.1)		259 (49.9)	446 (49.4)		194 (48.9)	313 (49.1)		89 (43.0)	201 (44.4)	
Blastocyst	50 (45.0)	94 (39.5)		143 (27.6)	232 (25.7)		111 (28.0)	155 (24.3)		75 (36.2)	159 (35.1)	
Endometrial thickness (mm)	9.1 ± 1.4	9.1 ± 1.5	0.873	9.4 ± 1.5	9.3 ± 1.4	0.18	9.5 ± 1.6	9.3 ± 1.4	0.125	9.1 ± 1.3	9.2 ± 1.4	0.52
Triple- line Endometrium (%)	108 (97.3)	213 (89.5)	0.012	489 (94.2)	809 (89.6)	0.003	371 (93.5)	579 (90.8)	0.124	203 (98.1)	398 (87.9)	0.000
Biochemical pregnancy rate (%)	92 (82.9)	149 (62.6)	0.000	290 (55.9)	470 (52)	0.164	215 (54.2)	322 (50.5)	0.248	151 (72.9)	270 (59.6)	0.001
Implantation rate (%)	102/198 (51.5)	156/418 (37.3)	0.000	288/895 (32.1)	435/1565 (27.7)	0.021	211/684 (30.8)	292/1112 (26.25)	0.035	164/362 (45.3)	218/783 (27.8)	0.000
Clinical pregnancy rate (%)	77 (69.4)	127 (53.4)	0.005	237 (45.7)	367 (40.6)	0.065	173 (43.6)	250 (39.2)	0.162	127 (61.4)	222 (49)	0.003
Ectopic pregnancy rate (%)	1 (0.9)	5 (2.1)	0.669	7 (1.3)	12 (1.3)	0.975	4 (1.0)	9 (1.4)	0.802	3 (1.4)	6 (1.3)	1.00
Early miscarriage (%)	7 (6.3)	15 (6.3)	0.541	30 (5.8)	50 (5.5)	0.848	19 (4.8)	40 (6.3)	0.317	16 (7.7)	23 (5.1)	0.18
Live birth (%)	65 (58.6)	99 (41.6)	0.003	183 (35.3)	285 (31.6)	0.153	137 (34.5)	187 (29.3)	0.079	100 (48.3)	180 (39.7)	0.039

^a^If the transferred embryo included at least one grade I cleavage embryo, one or two grade II cleavage embryos, and one or two blastocysts, they were classified as Cleavage I, Cleavage II, or blastocyst, respectively

^b^No record was collected for 76 patients

In the subgroup of patients with PCOS, the biochemical pregnancy, implantation, clinical pregnancy and live birth rates were higher in the GnRHa FET group than in the HRT FET group (82.9% vs. 62.6%, *p* < 0.001; 51.5% vs. 37.3%, *p* < 0.001; 69.4% vs. 53.4%, *p* = 0.005; and 58.6% vs. 41.6%, *p* = 0.003, respectively). In the subgroup of patients without PCOS, the GnRHa FET group produced a higher implantation (32.1% vs. 27.7%, *p* = 0.021), similar biochemical pregnancy (55.9% vs. 52%, *p* = 0.164), critically higher clinical pregnancy (45.7% vs. 40.6%, *p* = 0.065), and similar live birth rate (35.3% vs. 31.6%, *p* = 0.153), as compared to the HRT FET group. In the subgroup of patients with irregular menstruation, the biochemical pregnancy, implantation, clinical pregnancy and live birth rate were higher in the GnRHa FET group than in the HRT FET group (72.9% vs. 59.6%, *p* = 0.001; 45.3% vs. 27.8%, *p *< 0.001; 61.4% vs. 49%, *p* = 0.003;48.3% vs. 39.7%, *p* = 0.039;). However, in the subgroup of patients with regular menstruation, the GnRHa FET group produced a higher implantation rate (30.8% vs 26.2%, *p* = 0.035), but a similar biochemical pregnancy, clinical pregnancy and live birth rates (54.2% vs. 50.5%, *p* = 0.248; 43.6% vs. 39.2%, *p* = 0.162; 34.5% vs. 29.3%, *p* = 0.079), as compared to the HRT FET group.

Interestingly, we found that 1–3 dominant follicles grew in 37 patients14 days after GnRHa administration. The occurrence rate was 5.9%. The age of these patients was 34.1 ± 4.8 years. The endometrial thickness was 9.4 ± 1.5 mm on the day of progesterone administration with a rate of triple line pattern of 81.1%. After the transfer of 1.9 ± 0.3 embryos, the clinical pregnancy and live birth rates were 54.1% and 40.5%, respectively. The early abortion rate was 10.8% (Supplementary Tables 1 and 2).

## Discussion

With the widespread use of FET cycles, better protocols for endometrial preparation have been developed to improve pregnancy rates. The present study found that compared to the HRT FET cycles without GnRHa, the GnRHa FET group produced a significantly higher percentage of endometrium with a triple line pattern and improved pregnancy outcomes. Logistic regression analysis also showed that the GnRHa FET group was significantly associated with an increased chance of clinical pregnancy and live birth. These results demonstrate that the administration of a single dose of long-acting GnRHa in the early follicular phase of the same FET cycle can improve clinical outcomes, possibly by improving the receptivity of the endometrium. Meanwhile, after subgroup analysis, we found that age < 40 years, primary infertility, PCOS, and irregular menstruation are effective indications for endometrial preparation with GnRHa pretreatment in FET cycles. A relatively large amount of data provides evidence for the clinical application of this novel strategy in FET cycles. However, registered controlled trials are required to confirm the results of this study.

There is no consensus on which method of endometrial preparation in FET cycles is consistently better for pregnancy outcomes with or without GnRHa pretreatment. Negative results were observed between the two methods in some studies, that included patients with regular ovulation [14–18], regular menstrual cycles [5, 8] and functioning ovaries [7, 9, 11]. In contrast, compared to a natural or modified natural cycle protocol, retrospective data from 1391 cycles reported that the HRT protocol with GnRHa was associated with a higher live birth rate in the blastocyst-stage of the FET cycles [19]. It has been reported that pituitary suppression when initiated in the middle luteal phase of HRT cycles results in higher clinical pregnancy and live birth rates in patients with regular menstrual cycles than in those without prior GnRHa therapy [4]. Hebisha et al. reported that the administration of GnRHa for HRT FET during endometrial preparation increased the implantation and pregnancy rates in patients with undefined ovary functions [10]. In different studies, the time of GnRHa and the patients included were different. We proposed that this is one of the most important reasons for these inconsistent results.

In most published studies, short or long-acting GnRHa were administered during the mid-luteal phase for pretreatment. In the three aforementioned studies, short-acting GnRHa was administered daily stating in the middle luteal phase [4, 10, 19]. Le et al. administered medroxy-progesterone acetate for 10 days to induce menstruation and a half-dose of long-acting GnRHa on the third day of medroxy-progesterone acetate. The administration of exogenous estradiol was initiated on the third day of menstruation. They found that pregnancy outcomes were comparable to those observed in modified natural cycles [20]. Nekoo et al. and Prato et al. administered 3.75 mg of long-acting GnRHa at the mid-luteal phase (day 21) of the previous cycle, resulting in similar pregnancy rates between the HRT and GnRHa HRT FET cycles [5, 7].

Few studies have focused on the administration of long-acting GnRHa during the early follicular phase. In Qi’s study, 3.75 mg of long-acting GnRHa was injected on day 2 or 3 of menstruation with HRT 28 days later. They found that pregnancy outcomes were improved in patients with endometriosis and PCOS [21]. Xie et al. [22] administered 3.75 mg of long-acting GnRHa on day 3 of menstruation. After 28 days, estrogen and progesterone were administered as endometrial preparations. The data showed that the resultant clinical pregnancy and live birth rates were higher in the GnRHa HRT FET cycle than in the HRT FET cycle. Xu et al. recently reported that pretreatment with GnRHa failed to improve pregnancy outcomes in patients undergoing HRT-FET [23]. In our study, long-acting GnRHa was administered during the early follicular phase. It is easier for patients to recognize menstruation than luteal phase. Our results demonstrated that the administration of a single dose of long-acting GnRHa in the early follicular phase of the same FET cycle can be a novel strategy for endometrial preparation in FET cycles. The most effective interval between the GnRHa and estrogen administration is unknown. We found that an interval of 14 days between GnRHa and estrogen administration was sufficient for pretreatment and reduced the time the patient waited to start endometrial preparation. A shorter interval or administration of GnRHa and HRT together may also improve the pregnancy outcome of FET. This aspect warrants further investigation.

Pretreatment with GnRHa in FET cycles may has its indications for patients to improve pregnancy outcomes. Qi et al. found that pregnancy outcomes were improved in patients with endometriosis and PCOS after pretreatment with GnRHa [21]. However, one RCT reported that in patients with repeated implantation failure, short-term GnRHa from 21 day of menstruation did not increase pregnancy in subsequent HRT cycles [24]. In patients aged 38 years or older, Dong et al. failed to find a significant difference in pregnancy and live birth rates between the two groups [24]. In our study, after subgroup analysis, we found that pretreatment with GnRHa significantly increased clinical pregnancy and live birth rates in the subgroups of patients aged < 40 years, primary infertility, PCOS, and irregular menstruation, as compared to those in the HRT FET cycles. The results demonstrated that age < 40 years, primary infertility, PCOS, and irregular menstruation are effective indications for endometrial preparation with GnRHa pretreatment in FET cycles. However, this deserves further study RCTs.

Dominant follicles can be used for endometrial preparation after GnRHa administration because of their flare-up effect. ET was found to produce 54.1% of clinical pregnancy rate and 40.5% of live birth rate in this situation, suggesting that the downregulation of GnRHa did not affect embryo implantation when dominant follicles occurred; thus, the “cyst” need not to be punctured and the cycles need not to be cancelled or delayed under these conditions.

Clinical pregnancies and live births are associated with complex interactions between molecular pathways during fertilization, development and implantation of the embryo [25]. However, the mechanism by which GnRHa improves pregnancy outcomes in FET cycles remains unclear. First, the long desensitization buserelin protocol was used for ovarian stimulation in patients with endometriosis to improve endometrial receptivity and immunoregulation [26, 27]. During FET cycles, learn from ovarian stimulation, long-acting GnRHa then was used for pretreatment before endometrial preparation. The GnRH/ GnRHR system is expressed in the endometrium, ovaries, and human preimplantation embryos. The expression of this system supports its physiological regulatory role in the functioning of the corpus luteum [28], endometrial receptivity [29], trophoblast invasion [30], and embryo implantation [31, 32]. Fujii et al. continuously administered GnRHa during the luteal phase for14 days after oocyte retrieval in long protocol IVF [33]. The serum estradiol and progesterone concentrations on the day of embryo transfer and 7 days after oocyte retrieval were similar to those obtained using the long protocol alone. The implantation and live birth rates were significantly higher in the GnRHa group than in the control. These results suggested that GnRHa facilitates embryo implantation by enhancing luteal secretion. However, the results of our study cannot be explained on this basis. In our study, exogenous estrogen and progesterone were administered when there was no corpus luteum, except in the 37 patients who were undergoing ovulation. After 8 weeks of long-acting GnRHa administration, the pituitary gland began to recover its functions [34]. In our study, ET was performed at about 31–40 days after administering GnRHa, when the pituitary was in a state of suppression and the corpus luteum could not be stimulated. The GnRHa FET group produced a significantly higher percentage of endometrium with a triple line pattern, and improved pregnancy outcomes compared with the HRT FET cycles without GnRHa group. We propose that a possible mechanism could be the direct action of GnRHa in improving endometrial receptivity during FET cycles. In a murine model, ovarian stimulation decreased the endometrial expression of the integrin beta-3 subunit, leukaemia inhibitory factor, and the implantation rate during the implantation window. These effects were partially reversed by GnRHa administration. These results suggest that GnRHa plays an important role in improving the endometrial receptivity [29], thus supporting our hypothesis.

This study had several limitations. As this was a retrospective study and not a prospective randomized study, undetected biases may have occurred. Thus, we performed a logistic regression analysis to reduce bias. We did not evaluate important data, such as obstetrical and neonatal outcomes. A wide range of forms, doses, and routes of estrogen administration were utilized, which could potentially hinder the reproducibility of the results in future studies. Due to the small sample size, the clinical outcomes of GnRHa with dominant follicles without HRT were inconclusive. However, the reproductive outcomes were satisfactory in these patient groups.

## Conclusions

The administration of a single dose of long-acting GnRHa during the early follicular phase can improve the live birth rate in FET cycles. Age < 40 years, primary infertility, PCOS, and irregular menstruation are effective indications for endometrial preparation with GnRHa pretreatment in FET cycles. The mechanism underlying this process might rely on the direct effects of GnRHa on the regulation of endometrial receptivity. However, further RCT are required to validate the results of this study.

## Methods

### Study population and inclusion criteria

Patients who underwent FET October 2017 to March 2019 at a university-affiliated IVF center were retrospectively investigated. This study was approved by the Institutional Review Board (No.20190815) and conducted according to the principles of the Declaration of Helsinki. This manuscript conforms to the Enhancing the Quality and Transparency of Health Research (EQUATOR) network guidelines. The inclusion criterias were as follows: (1) patients who underwent FET cycles with GnRHa pretreatment (GnRHa FET) and HRT FET cycles without GnRH agonist (HRT) as the control group at the corresponding period for endometrial preparation according to electronic records; (2) patients aged 45-years old or less; and (3) at least one grade I, II, or blastocyst embryo transferred. Cleavage stage embryos were grade with Istanbul consensus 2011 and blastocysts were graded with the Gardner scoring system; (4) endometrial thickness on the day of progesterone supplementation was ≥ 6.5 mm. Patients with intrauterine adhension, untreated hydrosalpinx or severe adenomyosis were excluded from this study. Patients with severe endometriosis including adenomyosis received two and more GnRHa treatments were excluded.

### Endometrial preparation protocols

#### HRT FET without GnRH agonist

On the third day of menstruation, ultrasonography was performed to exclude the patients with functional ovarian cysts in the control group. If patients were amenorrhea, 6–7 days of oral dydrogesterone or progesterone capsules were used to induce menstruation. Estradiol valerate ((Progynova, Bayer) or a white estradiol tablet (Femoston, Abbott) was administered at an initial dose of 2 mg bid, which was increased to 3-4 mg bid as necessary. Transdermal estradiol gel (Oestrogel, Besins, France) was administered at an initial dose of 1.5–2 slide calipers bid. Estrogen was administered for at least 12 days. The endometrial thickness was monitored. If it reached at least 6.5 mm and no dominant follicles were found, vaginal micronized progesterone supplementations were administered at a dose of 400 mg bid for endometrial conversion. Cleavage embryos were transferred on the fourth day of progesterone administration and blastocyst embryos were transferred on the sixth day.

#### FET with GnRH agonist

On the second or third day of menstruation, ultrasonography was performed to rule out the presence of functional ovarian cysts in the experimental groups. Then, 3.75 mg of leuprorelin was administered. If no dominant follicles were observed on ultrasound 14 days later, estrogen and progesterone were administered, and the embryo was transferred as described above. If dominant follicles were found, HMG was used to promote the development of the follicles as necessary, after which ovulation was triggered by HCG with the thickness of the endometrium reaching at least 6.5 mm. After ovulation or 2 days after HCG injection, progesterone was added for endometrial conversion. Cleavage and blastocyst embryos were transferred on the third and fifth days post-ovulation or on the fifth and seventh days after HCG administration, respectively.

#### Outcomes

Twelve days after embryo transfer, the concentration of HCG in the serum was tested. If the pregnancy test was positive 28–35 days after ET, transvaginal ultrasonography was performed to confirm clinical pregnancy. Luteal support was continued for up to two months after FET. The live birth rate was the primary outcome considered in this study. The secondary outcomes were the endometrial thickness on the day of progesterone supplementation and clinical pregnancy rate. Early miscarriages were defined as spontaneous clinical pregnancy loss prior to 12 weeks of gestation. Implantation rate was defined as the ratio of the number of gestational sacs over the number of transferred embryos. Clinical pregnancy was defined as a visible yolk sac on ultrasonography.

### Statistical analysis

Statistical analysis was performed using the SPSS 23.0 (IBM, Armonk, NY, USA). Measurement data are presented as the mean ± standard deviation (SD) and were analyzed using Student’s t-test or Fisher’s exact test where appropriate. Enumeration data are presented as percentages (%) and were analyzed using the chi-square test. Statistical significance was set *p* < 0.05. Binary logistic regression analysis was used to assess the association between the GnRHa level and clinical pregnancies. We calculated odds ratios (OR) and 95% confidence interval (CI). Hosmer–Lemeshow test was conducted to assess the overall quality of the final model. Subgroups analysis we performed between age < 40 years with ≥ 40 years, primary with secondary infertility, PCOS with no PCOS, and regular with irregular menstruation respectively.

### Supplementary Information

**Additional file 1:** **Supplementary Table 1.** Characteristics of patients in GnRHa+ovulation vs. GnRHa+HRT. **Supplementary Table 2.** Outcomes of FET for GnRHa+ovulation vs. GnRHa+HRT.

## Data Availability

The raw data supporting the conclusions of this article will be made available by the authors without undue reservation.

## References

[1] Groenewoud ER, Cantineau AE, Kollen BJ, Macklon NS, Cohlen BJ (2013). What is the optimal means of preparing the endometrium in frozen-thawed embryo transfer cycles? A systematic review and meta-analysis. Hum Reprod Update.

[2] Ghobara T, Gelbaya TA, Ayeleke RO (2017). Cycle regimens for frozen-thawed embryo transfer. Cochrane Database Syst Rev.

[3] Kupka MS, D'Hooghe T, Ferraretti AP, European IVFMC, European Society of Human R, Embryology, (2016). Assisted reproductive technology in Europe, 2011: results generated from European registers by ESHRE. Hum Reprod..

[4] El-Toukhy T, Taylor A, Khalaf Y, Al-Darazi K, Rowell P, Seed P (2004). Pituitary suppression in ultrasound-monitored frozen embryo replacement cycles. A randomised study. Hum Reprod.

[5] Azimi Nekoo E, Chamani M, Shahrokh Tehrani E, Hossein Rashidi B, Davari Tanha F, Kalantari V (2015). Artificial Endometrial Preparation for Frozen-Thawed Embryo Transfer with or without Pretreatment with Depot gonadotropin Releasing Hormone Agonist in Women with Regular Menses. J Fam Reprod Health.

[6] Samsami A, Chitsazi Z, Namazi G (2018). Frozen thawed embryo transfer cycles; A comparison of pregnancy outcomes with and without prior pituitary suppression by GnRH agonists: An RCT. Int J Reprod Biomed.

[7] Dal Prato L, Borini A, Cattoli M, Bonu MA, Sciajno R, Flamigni C (2002). Endometrial preparation for frozen-thawed embryo transfer with or without pretreatment with gonadotropin-releasing hormone agonist. Fertil Steril.

[8] Kang J, Park J, Chung D, Lee SH, Park EY, Han KH (2018). Comparison of the clinical outcome of frozen-thawed embryo transfer with and without pretreatment with a gonadotropin-releasing hormone agonist. Obstet Gynecol Sci.

[9] Simon A, Hurwitz A, Zentner BS, Bdolah Y, Laufer N (1998). Transfer of frozen-thawed embryos in artificially prepared cycles with and without prior gonadotrophin-releasing hormone agonist suppression: a prospective randomized study. Hum Reprod.

[10] Hebisha SA, Adel HM (2017). GnRh agonist treatment improves implantation and pregnancy rates of frozen-thawed embryos transfer. J Obstet Gynaecol India.

[11] Movahedi S, Aleyasin A, Agahosseini M, Safdarian L, Abroshan S, Khodaverdi S (2018). Endometrial preparation for women undergoing embryo transfer frozen-thawed embryo transfer with and without pretreatment with gonadotropin releasing hormone agonists. J Fam Reprod Health.

[12] van de Vijver A, Polyzos NP, Van Landuyt L, De Vos M, Camus M, Stoop D (2014). Cryopreserved embryo transfer in an artificial cycle: is GnRH agonist down-regulation necessary?. Reprod Biomed Online.

[13] Luo L, Chen M, Wen Y, Zhang L, Zhou C, Wang Q (2021). Pregnancy outcome and cost-effectiveness comparisons of artificial cycle-prepared frozen embryo transfer with or without GnRH agonist pretreatment for polycystic ovary syndrome: a randomised controlled trial. BJOG.

[14] al-Shawaf T, Yang D, al-Magid Y, Seaton A, Iketubosin F, Craft I (1993). Ultrasonic monitoring during replacement of frozen/thawed embryos in natural and hormone replacement cycles. Hum Reprod.

[15] Queenan JT, Veeck LL, Seltman HJ, Muasher SJ (1994). Transfer of cryopreserved-thawed pre-embryos in a natural cycle or a programmed cycle with exogenous hormonal replacement yields similar pregnancy results. Fertil Steril.

[16] Tanos V, Friedler S, Zajicek G, Neiger M, Lewin A, Schenker JG (1996). The impact of endometrial preparation on implantation following cryopreserved-thawed-embryo transfer. Gynecol Obstet Invest.

[17] Gelbaya TA, Nardo LG, Hunter HR, Fitzgerald CT, Horne G, Pease EE (2006). Cryopreserved-thawed embryo transfer in natural or down-regulated hormonally controlled cycles: a retrospective study. Fertil Steril.

[18] Mounce G, McVeigh E, Turner K, Child TJ (2015). Randomized, controlled pilot trial of natural versus hormone replacement therapy cycles in frozen embryo replacement in vitro fertilization. Fertil Steril.

[19] Hill MJ, Miller KA, Frattarelli JL (2010). A GnRH agonist and exogenous hormone stimulation protocol has a higher live-birth rate than a natural endogenous hormone protocol for frozen-thawed blastocyst-stage embryo transfer cycles: an analysis of 1391 cycles. Fertil Steril.

[20] Le QV, Abhari S, Abuzeid OM, DeAnna J, Satti MA, Abozaid T (2017). Modified natural cycle for embryo transfer using frozen-thawed blastocysts: a satisfactory option. Eur J Obstet Gynecol Reprod Biol.

[21] Qi Q, Luo J, Wang Y, Xie Q (2020). Effects of artificial cycles with and without gonadotropin-releasing hormone agonist pretreatment on frozen embryo transfer outcomes. J Int Med Res.

[22] Xie D, Chen F, Xie SZ, Chen ZL, Tuo P, Zhou R (2018). Artificial cycle with or without a depot gonadotropin-releasing hormone agonist for frozen-thawed embryo transfer: an assessment of infertility type that is most suitable. Current medical science.

[23] Xu J, Li SZ, Yin MN, Liang PL, Li P, Sun L (2021). Endometrial preparation for frozen-thawed embryo transfer with or without pretreatment with GnRH agonist: a randomized controlled trial at two centers. Front Endocrinol (Lausanne).

[24] Davar R, Dashti S, Omidi M (2020). Endometrial preparation using gonadotropin-releasing hormone agonist prior to frozen-thawed embryo transfer in women with repeated implantation failure: an RCT. Int J Reprod Biomed.

[25] Lagana AS, Uccella S, Chiantera V, Garzon S (2022). Molecular biology of human fertility: stepping towards a Tailored approach. Int J Mol Sci.

[26] Salamun V, Verdenik I, Lagana AS, Vrtacnik-Bokal E (2018). Should we consider integrated approach for endometriosis-associated infertility as gold standard management? Rationale and results from a large cohort analysis. Arch Gynecol Obstet.

[27] Mikus M, Goldstajn MS, Brlecic I, Dumancic S, Lagana AS, Chiantera V (2022). CTLA4-linked autoimmunity in the pathogenesis of endometriosis and related infertility: a systematic review. Int J Mol Sci.

[28] Kyrou D, Kolibianakis EM, Fatemi HM, Tarlatzi TB, Devroey P, Tarlatzis BC (2011). Increased live birth rates with GnRH agonist addition for luteal support in ICSI/IVF cycles: a systematic review and meta-analysis. Hum Reprod Update.

[29] Ruan HC, Zhu XM, Luo Q, Liu AX, Qian YL, Zhou CY (2006). Ovarian stimulation with GnRH agonist, but not GnRH antagonist, partially restores the expression of endometrial integrin beta3 and leukaemia-inhibitory factor and improves uterine receptivity in mice. Hum Reprod.

[30] Iwashita M, Kudo Y, Shinozaki Y, Takeda Y (1993). Gonadotropin-releasing hormone increases serum human chorionic gonadotropin in pregnant women. Endocr J.

[31] Maggi R, Cariboni AM, Marelli MM, Moretti RM, Andre V, Marzagalli M (2016). GnRH and GnRH receptors in the pathophysiology of the human female reproductive system. Hum Reprod Update.

[32] Casan EM, Raga F, Polan ML (1999). GnRH mRNA and protein expression in human preimplantation embryos. Mol Hum Reprod.

[33] Fujii S, Sato S, Fukui A, Kimura H, Kasai G, Saito Y (2001). Continuous administration of gonadotrophin-releasing hormone agonist during the luteal phase in IVF. Hum Reprod.

[34] Broekmans FJ, Bernardus RE, Broeders A, Berkhout G, Schoemaker J (1993). Pituitary responsiveness after administration of a GnRH agonist depot formulation: Decapeptyl CR. Clin Endocrinol.

