# Supplementary material for: Pretreatment with a long-acting GnRH agonist for frozen-thawed embryo transfer cycles: how to improve live birth?
Source: J Ovarian Res. 2023 Sep 25;16:197. doi: 10.1186/s13048-023-01277-0 (PMC10518919; doi:10.1186/s13048-023-01277-0)
Supplement: Supplementary file 1 — Additional file 1: Supplementary Table 1. Characteristics of patients in GnRHa+ovulation vs. GnRHa+HRT. Supplementary Table 2. Outcomes of FET for GnRHa+ovulation vs. GnRHa+HRT. [file 13048_2023_1277_MOESM1_ESM.docx]

**Supplementary Table 1.** Characteristics of patients in GnRHa+ovulation vs. GnRHa+HRT

| **Characteristics** | **GnRHa+ovulation (n=37)** | **GnRHa+HRT**  **(n=593)** | ***p*** |
| --- | --- | --- | --- |
| Age | 34.1±4.8 | 32.0±5.2 | 0.015 |
| Duration of infertility | 6.1±4.4 | 4.7±3.5 | 0.071 |
| Type of infertility |  |  | 0.015 |
| Primary | 11 (29.7) | 299 (50.4) |  |
| Secondary | 26 (70.3) | 294 (49.6) |  |
| Number of pregnancies | 1.6±1.4 | 1.1±1.4 | 0.058 |
| PCOS（%） | 2 (5.4) | 109 (18.4) | 0.044 |
| DOR（%） | 7 (18.9) | 79 (13.3) | 0.180 |
| endometriosis（%） | 1 (2.7) | 22 (3.7) | 1.000 |
| Percentage of scared uterus | 11(29.7) | 91 (15.3) | 0.021 |
| Number of transferred embryos | 1.9±0.3 | 1.7±0.4 | 0.026 |
| Grade of transferred embryos |  |  | 0.225 |
| Cleavage I | 11 (29.7) | 127 (21.4) |  |
| Cleavage II | 19 (51.4) | 280 (47.2) |  |
| Blastocyst | 7 (18.9) | 186 (31.4) |  |

GnRHa, gonadotropin-releasing hormone agonist; HRT, hormone replacement treatment; PCOS, polycystic ovary syndrome; DOR, decreased ovarian reserve.

**Supplementary Table 2.** Outcomes of FET for GnRHa+ovulation vs. GnRHa+HRT.

| **Outcome** | **GnRHa+ovulation (n=37)** | **GnRHa+HRT**  **(n=593)** | ***p*** |
| --- | --- | --- | --- |
| Endometrial thickness | 9.4±1.5 | 9.3±1.5 | 0.215 |
| Endometrial pattern |  |  | 0.002 |
| Three line | 30 (81.1) | 567 (95.6) |  |
| Non-three line | 7 (18.9) | 26 (4.4) |  |
| Biochemical pregnancy (%) | 23 (62.2) | 359 (60.5) | 0.845 |
| Implantation (%) | 23/69 (33.3) | 366/1024 (35.7) | 0.75 |
| Clinical pregnancy (%) | 20 (54.1) | 294 (49.6) | 0.597 |
| Ectopic pregnancy (%) | 1 (2.7) | 7 (1.2)^a^ | 0.422 |
| Early abortion (%) | 4/37 (10.8) | 33/593 (5.6) | 0.264 |
| Live birth (%) | 15 (40.5) | 248 (39.3) | 0.88 |

Note FET, frozen-thawed embryo transfer; GnRHa, gonadotropin-releasing hormone agonist; HRT, hormone replacement treatment

^a^Included two heterotopic pregnancies.
